# The chick somitogenesis oscillator is arrested before all paraxial mesoderm is segmented into somites

**DOI:** 10.1186/1471-213X-10-24

**Published:** 2010-02-25

**Authors:** Gennady Tenin, David Wright, Zoltan Ferjentsik, Robert Bone, Michael J McGrew, Miguel Maroto

**Affiliations:** 1 Division of Cell and Developmental Biology, College of Life Sciences, University of Dundee, Dow Street, Dundee, UK; 2 The Roslin Institute and Royal Dick School of Veterinary Studies, University of Edinburgh, Roslin, UK

## Abstract

**Background:**

Somitogenesis is the earliest sign of segmentation in the developing vertebrate embryo. This process starts very early, soon after gastrulation has initiated and proceeds in an anterior-to-posterior direction during body axis elongation. It is widely accepted that somitogenesis is controlled by a molecular oscillator with the same periodicity as somite formation. This periodic mechanism is repeated a specific number of times until the embryo acquires a defined specie-specific final number of somites at the end of the process of axis elongation. This final number of somites varies widely between vertebrate species. How termination of the process of somitogenesis is determined is still unknown.

**Results:**

Here we show that during development there is an imbalance between the speed of somite formation and growth of the presomitic mesoderm (PSM)/tail bud. This decrease in the PSM size of the chick embryo is not due to an acceleration of the speed of somite formation because it remains constant until the last stages of somitogenesis, when it slows down. When the chick embryo reaches its final number of somites at stage HH 24-25 there is still some remaining unsegmented PSM in which expression of components of the somitogenesis oscillator is no longer dynamic. Finally, we identify a change in expression of retinoic acid regulating factors in the tail bud at late stages of somitogenesis, such that in the chick embryo there is a pronounced onset of *Raldh2 *expression while in the mouse embryo the expression of the RA inhibitor *Cyp26A1 *is downregulated.

**Conclusions:**

Our results show that the chick somitogenesis oscillator is arrested before all paraxial mesoderm is segmented into somites. In addition, endogenous retinoic acid is probably also involved in the termination of the process of segmentation, and in tail growth in general.

## Background

Somitogenesis is the earliest sign of segmentation in the developing vertebrate embryo [1-3]. During this process vertebrate embryos generate transitory structures called somites that later in development give rise to the vertebral column, most of the skeletal musculature and much of the dermis [4]. This process starts very early soon after gastrulation has initiated and proceeds in an anterior-to-posterior direction during body axis elongation. The elongation of the body axis of the vertebrate embryo has been traditionally divided into two phases termed primary and secondary body formation [5,6]. During the first phase the somites and other types of mesoderm are derived from cells that have traversed the primitive streak (in amniotes) or its equivalent (in anamniote vertebrates). Fate mapping analyses have shown that the primitive streak contains distinct stem cell populations in specific domains along the antero-posterior axis of the streak [7-9]. During secondary body formation the tail bud is the source of somitic mesoderm precursors [10,11]. At first considered to be a homogeneous blastema of tissue, lineage analysis has since shown that distinct stem cell populations also exist in specific domains within the tail bud, as proposed by Pasteels [12], and they are capable of contributing to multiple tissue types [5,13-20].

During axis elongation two parallel bands of paraxial mesoderm tissue known as the unsegmented or presomitic mesoderm (PSM) migrate out from the primitive streak (or tail bud) and come to lie alongside the notochord. Groups of cells at the most rostral end of each PSM bud off with a remarkable periodicity and synchronisation as an epithelial sphere of cells to form the new somite. It is widely accepted that this process is controlled by a molecular oscillator [21] that drives periodic waves of gene expression caudo-rostrally through the PSM with the same periodicity as somite formation. In fact, in recent years the community has reported the discovery and characterization of a considerable number of these so-called clock genes in a variety of vertebrate species, all of which are components of the Notch, Wnt or FGF pathways [22-24]. This periodic mechanism is repeated a specific number of times until the embryo acquires a defined species-specific final number of somites at the end of the process of axis elongation. This final number of somites varies widely between vertebrate species: 38-39 somites in the human embryo, 51-53 in the chick embryo, 62-65 in the mouse embryo and several hundred somites in snake embryos of different species [25].

How termination of the process of somitogenesis, and tail bud growth in general, are determined is still unknown. Several mechanisms have been proposed to contribute including an overall control of somite number exerted by the embryo as a whole [26], a localised cell death overtaking the process [26,27], inadequacies in the local environment or in the cells themselves [28] or suppression of the epithelial-mesenchymal transition required for gastrulation-like movements [29]. In this study we analysed the anatomy of the tail bud at the end of the process and went on to investigate this tissue with respect to apoptosis, the somitogenesis oscillator and the regulation by endogenous retinoic acid (RA) of factors associated to the growth and proliferation of the precursor stem cells.

## Results

### PSM elongation is not happening at the same speed as somite formation

**Figure 1:**
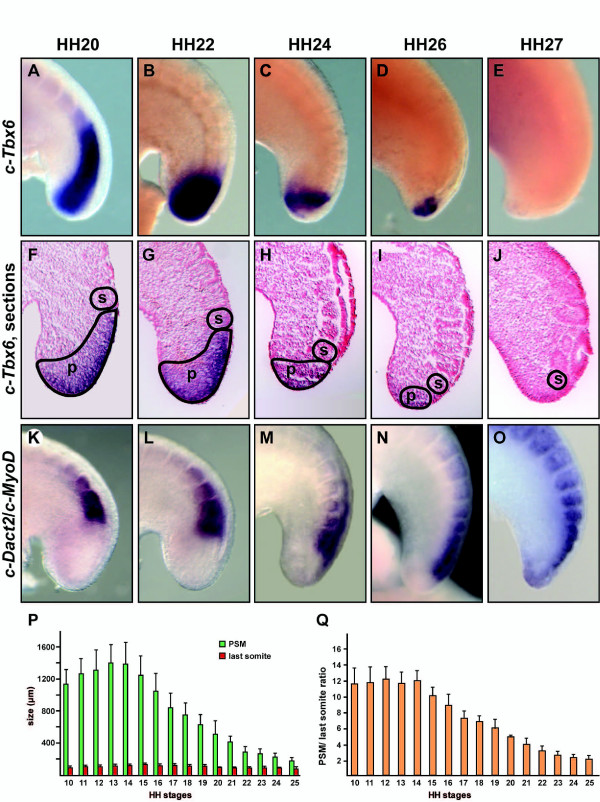
**Regression of the PSM domain in the chick tail bud**. Lateral view of whole-mount chick tail buds at (A, F, K) HH stage 20, (B, G, L) stage 22, (C, H, M) stage 24, (D, I, N) stage 26 and (E, J, O) stage 27 analysed by *in situ *hybridisation with (A-J) *c-Tbx6 *and (K-O) *c-Dact2*/*c-MyoD *showing the position of the PSM or the first somite, respectively. (F-J) Sections of tail buds stained with the *c-Tbx6 *probe; the borders of the PSM domain (p) and the last somite (s) are demarcated. (P) Bar chart showing the size of the PSM (green bars) and the size of the last somite (red bars) at HH stages 10-25. (Q) Bar chart showing the PSM/last somite ratio during the same window of development calculated separately for each embryo and then the average ratio was calculated for each stage. Error bars represent standard deviation.

To investigate the cessation of the process of somitogenesis in the chick tail bud it is important first to know if indeed, as previously proposed, the somites extend to the tip of the tail at HH stage 22 [30] or whether this is not the case and there remains some unsegmented PSM [26,28]. The somites extending close to the tip would indicate there is an imbalance between the speed of axis elongation and the recruitment of new cells to become part of the PSM tissue, while if the paraxial mesoderm remains unsegmented this would indicate that the somitogenesis oscillator stops producing somites at a specific stage of development. Thus, we collected chick embryos ranging in development from HH stage 20 to 27 and stained them by *in situ *hybridisation either with *c-Tbx6*, a probe specific for the non-segmented PSM (n = 14, Figure 1A-J), or with *c-Dact2*/*c-MyoD *a combination of probes to specifically label the most recently formed somites (n = 21, Figure 1K-O). As development proceeds we observed that the domain of *c-Tbx6 *expression in the non-segmented mesoderm becomes progressively smaller in the tail bud until it disappears at HH stage 26-27. Concurrently, we detected the domain of *c-Dact2*/*c-MyoD *expression in the last formed somites gradually moving closer to the end of the tail bud until it almost reached the tip at stage 26-27 HH. We concluded from these results that in fact at the end of somitogenesis the somites nearly reach the tip of the tail bud. We then measured the length of the PSM, the diameter of the last formed somite and the ratio PSM length versus last somite diameter in a large number of embryos ranging from HH stage 10-24 (n = 105). These graphical representations show that until HH stage 14 both parameters increase slightly and then decrease as development proceeds further (Figure 1P, Q; [31]) indicating that from HH stage 14 the elongation of the chick PSM tissue is not happening at the same speed as somite formation. We reasoned that the observed imbalance could be produced by an acceleration in the speed of somite formation or the result of a progressive reduction in the growth/number of precursor stem cells due to changes in their proliferation status and/or apoptosis. We investigated if either of these mechanisms indeed contributes to the cessation of this process.

### Deceleration of the speed of somite formation

**Figure 2:**
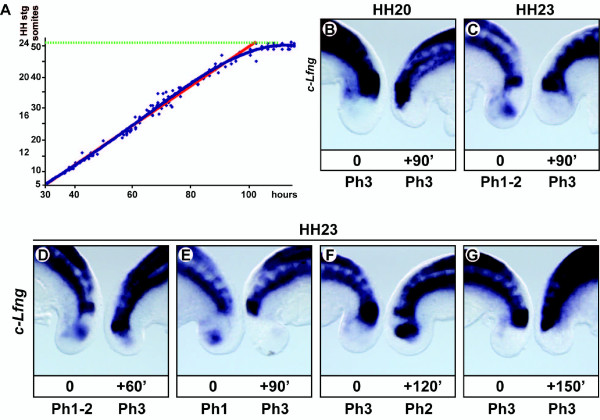
**Time course of somite number during chick somitogenesis. Extension of the somite formation period**. (A) Graph plotting increasing time intervals along the *x *axis and increasing number of somites and the corresponding HH stage of chick development along the *y *axis. The red line is a theoretical line showing the progression if the speed of somitogenesis were to remain constant at 90 minutes throughout development. Each blue dot corresponds to an individual sample/embryo. The blue line is a best fit line. Dotted green line demarcates the average final number of somites. (B-G) Fix and culture analysis using the half embryo system using the caudal region of a chick embryo. Left half samples were immediately fixed whereas right half samples were cultured *in vitro *for different periods of time and then the expression of *c-Lfng *was analysed in the two halves by *in situ *hybridisation. (B) HH stage 20 and (C) stage 23 samples cultured for 90 minutes. (D-G) HH stage 23 samples were cultured for (D) 60 minutes, (E) 90 minutes, (F) 120 minutes and (G) 150 minutes.

To determine if an acceleration in the speed of somitogenesis at late stages contributes to the loss of PSM tissue and the termination of somite formation we analysed a large batch (n = 105) of HH stage 10 embryos incubated for specific periods of time and then recording the number somites formed (Figure 2A). A regression analysis allowed us to conclude that at least until approx. HH stage 21 the periodicity of somite formation remains constant at 90 minutes. According to the results only the speed of formation of the last 5-8 somites is different to the rest. Interestingly their formation pace does not seem to get faster but in fact gets progressively slower. To confirm the existence of this deceleration in the speed of somite formation we performed an *in vitro *experiment in which the tail of a chick embryo is split in two halves, one half is immediately fixed and the second half is cultured for 90 minutes, and then both samples are analysed by *in situ *hybridisation for the expression of a clock gene, such as the Notch target gene *c-Lfng*. When this analysis was performed using samples of early stages of somitogenesis, such as HH stage 19-20, the two halves displayed the same pattern of expression of *c-Lfng *and a new somite was apparent in the cultured half, consistent with a periodicity of somite formation of 90 minutes (n = 15, Figure 2B; [32]). However, when the same experiment was repeated with late stages of somitogenesis, such as HH stage 23, the two halves displayed different patterns of expression of *c-Lfng *suggesting that the cultured half has not been able to complete the whole cycle in 90 minutes (n = 9, Figure 2C, [33]). To confirm this result we repeated the experiment and cultured the samples for different periods of time ranging from 60 to 150 minutes (n = 42). We found that the two HH stage 23 halves displayed the same pattern of expression only after one half had been cultured for 150 minutes longer than the other (Figure 2D-G), which is therefore the time required to make a new somite at this stage of development. Thus, the imbalance between the speed of axis elongation and the recruitment of new cells to become part of the non-segmented mesoderm cannot be due to acceleration in the speed of somitogenesis.

### The somitogenesis oscillator is arrested at late stages of development

**Figure 3:**
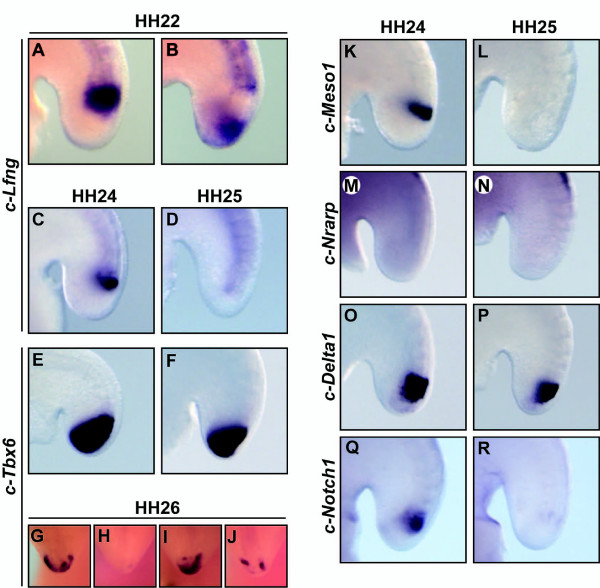
**Components of the Notch-based somitogenesis oscillator are downregulated in the chick tail bud at HH stage 25**. Lateral views of chick embryos of (A-B) HH stage 22, (C, E, K, M, O, Q) stage 24 and (D, F, L, N, P, R) stage 25, and (G-J) frontal views of chick embryos of HH stage 26 analysed by *in situ *hybridisation to show the expression of (A-D) *c-Lfng*, (E-J) *c-Tbx6*, (K, L) *c-Meso1*, (M, N) *c-Nrarp*, (O, P) *c-Delta1 *and (Q, R) *c-Notch1*. From HH stage 24 the expression of components of the somitogenesis oscillator becomes restricted to the rostral region of the PSM and then at HH stage 25 their expression is downregulated.

Our results also showed that chick embryos produce a total of 51-53 somites that in most cases are already formed by HH stage 24-25 (dotted green line in Figure 2A). Curiously, at those stages there is still a remaining population of *c-Tbx6 *positive PSM cells in the tail bud (Figure 1C, D, H, I), suggesting that the last PSM tissue probably does not contribute to the formation of new somites. To investigate this possibility we decided to explore the activity of the somitogenesis oscillator by examining the expression of Notch regulated cyclic genes, such as *c-Lfng*, in batches of embryos at several developmental stages to see if different patterns of expression could be observed, which would be indicate that the oscillator was still operational. At HH stage 22 (n = 10), when the embryo is still forming somites, we found clear different patterns of expression of *c-Lfng *in the PSM (Figure 3A, B), consistent with the idea that at this stage the expression is still dynamic. Surprisingly, the situation was different at later stages and thus at HH stage 24 (n = 10) *c-Lfng *expression was present but restricted to the rostral region of the PSM (Figure 3C), whereas at HH stage 25 (n = 7) *c-Lfng *had disappeared completely from the PSM (Figure 3D). As mentioned above, at these stages of development there is still some *Tbx6*-positive PSM tissue (Figure 3E, F). We then examined the expression of two more genes regulated by Notch activity, *c-Meso1 *and *c-Nrarp*. *Meso1 *is the homologue of mouse *Mesp2*, a critical factor for the formation of the epithelial somite [34] normally expressed in the rostral region of the PSM. At HH stage 24 *Meso1 *was still present in the rostral PSM but by HH stage 25 the expression was lost (n = 10, Figure 3K, L). *Nrarp *is another Notch-based cyclic gene [35,36] and, surprisingly, it is no longer expressed in the PSM at either of these two stages of development (n = 9, Figure 3M, N). Lastly, we examined the expression of the Notch ligand *Delta1 *and the receptor *Notch1 *and found that whereas *Delta1 *(n = 9) transcripts appear to remain strong at least in the median and rostral PSM, *Notch1 *(n = 7) becomes downregulated (Figure 3O-R). Thus, from these expression patterns of the different Notch components, we conclude that at HH stage 24-25 Notch activity implicated in the mechanism of the oscillator in the PSM becomes first restricted to the rostral region of the PSM and is then abolished from the tissue.

Furthermore, we found that the expression of *c-Tbx6 *in the tail bud of different HH stage 26 chick embryos seems to diminish very irregularly until it disappears (n = 10, Figure 3G-J), in contrast to the organized pattern in which *Tbx6 *expression is normally downregulated in PSM cells as they become integrated into a new somite (Figure 1A-J). We tested if this irregular downregulation of *c-Tbx6 *could be temporally related to apoptosis, a process reported previously to be affecting the tail bud region [26,27,37-39]. To that end we performed TUNEL staining in chick embryos from HH stage 13 to stage 27 (n = 34). This analysis clearly showed the existence of a localized domain of apoptosis in the chick tail bud affecting the whole area until HH stage 20-21 [Additional File 1] and then again at HH stage 25-26 [Additional File 1], which indeed could be a contributing factor to the elimination of the remaining *Tbx6 *positive PSM tissue that does not segment into somites.

### Onset of *c-Raldh2 *expression and retinoic activity in the chick tail bud

**Figure 4:**
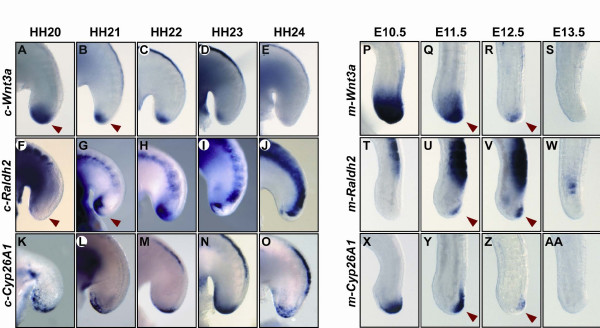
**Onset of *c-Raldh2 *expression in the chick tail bud from HH stage 21**. Lateral view of whole-mount chick tail buds at (A, F, K) HH stage 20, (B, G, L) stage 21, (C, H, M) stage 22, (D, I, N) stage 23 and (E, J, O) stage 24 analysed by *in situ *hybridisation with (A-E) *c-Wnt3a*, (F-J) *c-Raldh2 *and (K-O) *c-Cyp26A1 *to show their expression in the tail bud region. Red arrowheads show that from HH stage 20 to stage 21 there is a severe downregulation of *c-Wnt3a *simultaneous to an onset of *c-Raldh2 *in the tail bud. Lateral view of mouse tail buds at (P, T, X) E10.5, (Q, U, Y) E11.5, (R, V, Z) E12.5 and (S, W, AA) E13.5 analysed by *in situ *hybridisation using (P-S) *m-Wnt3a*, (T-W)*m-Raldh2*, and (X-AA)*m-Cyp26A1 *probes to show their expression in the tail bud region. Red arrowheads show that from E10.5 to E13.5 there is a severe downregulation of *m-Wnt3a *simultaneous to an onset of *m-Raldh2 *in the tail bud and a downregulation of *m-Cyp26A1*.

As an alternative or additional possible mechanism contributing to the cessation of somite formation and tail bud growth we searched for patterns of expression that would be consistent with changes in the proliferation status of the precursor population. A number of reports have previously shown that Wnt3a is implicated in the generation of mesodermal tissues within the developing primitive streak and tail bud, and it plays a pivotal role in the normal development and proliferation of these structures [40-44]. In the absence of Wnt3a the growth of caudal structures is seriously impaired, as seen in the *vestigial tail *(*vt*) mutant embryo, a hypomorphic allele with severely downregulated expression of *Wnt3a *in the tail bud that suffers severe caudal agenesis [40]. Similarly, when *Wnt3a *expression is downregulated by exposure to ectopic retinoic acid (RA) the embryo displays axial truncations [45-48]. A comparable phenotype is also observed in homozygous embryos of *Cyp26A1*-/- because in the absence of this RA-degrading enzyme the tail bud becomes exposed to endogenous RA produced by the somites, which acts to downregulate Wnt3a in the tail bud [49-51]. Thus, we decided to re-examine by *in situ *hybridisation the temporal and spatial expression profile of members of Wnt and RA signalling pathways in the tail bud of the chick embryo at HH stage 20-24. Our analysis shows that *c-Wnt3a *is expressed in a broad area of the tail bud until HH stage 20, whereupon its expression is dramatically reduced to the very tip until it disappears at HH stage 24 (n = 38, Figure 4A-E). A similar profile of expression is detected for the Wnt regulated gene, *c-Fgf8 *(data not shown). When we checked the expression of *c-Raldh2 *(n = 27), which encodes an RA-producing enzyme, we found that it is not expressed in the tail bud at early stages (Figure 4F). However, from HH stage 21 we observed a previously unreported onset of *c-Raldh2 *expression in the chick tail bud, which remained in this domain until later stages (Figure 4G-J). The transcripts for the enzyme c-Cyp26A1 involved in the catabolism and inactivation of RA were expressed in the tail bud at all stages analysed (n = 16, Figure 4K-O). The onset of *Raldh2 *in the tail bud is an intriguing observation because it suggests that from HH stage 21 the chick tail bud might be exposed to endogenous RA produced in that very tissue. As mentioned above, there are examples showing there is a correlation between levels of RA and the expression of *Fgf8 *and *Wnt3a *[45-48,52-54].

**Figure 5:**
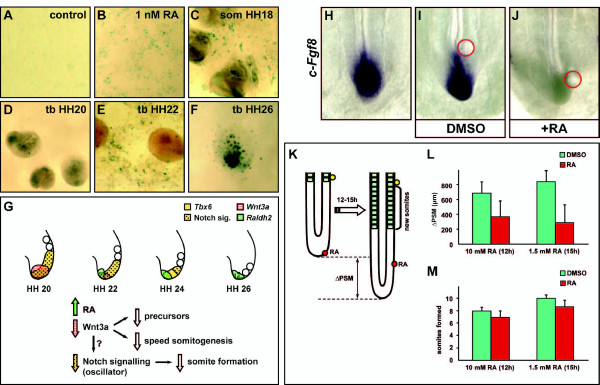
***Wnt3a *diminution and RA activity in the chick tail bud**. RA detection using F9-1.8 cells cultured in (A) RA-free media, (B) with 1 nM RA or co-cultured with (C) somitic tissue, (D) tail bud tissue from a HH stage 20, (E) HH stage 22 and (F) HH stage 26. (G) Schematic representation. From HH stage 21 there is an onset of *c-Raldh2 *in the proximity of the precursor cells located in the chick tail bud. This local RA activity is likely to negatively affect the expression of *c-Wnt3a*, which subsequently affects the growth of the precursor population and the speed of somite formation, and might also impinge on the observed loss of Notch activity associated with the somitogenesis oscillator. (H-J) Dorsal view of chick embryo analysed by *in situ *hybridisation using a *c-Fgf8 *probe. (H) control, (I) exposure to DMSO and (J) 1.5 mM RA soaked beads. (K) Scheme of experiment with RA soaked beads. Red circle shows the position of the DMSO/RA soaked bead in the tail bud; yellow circle represents the DMSO reference bead used as a means of measuring PSM size before and after treatment. (L-M) 1.5-10 mM RA soaked beads in the tail bud (L) slow down the growth of the PSM and (M) reduces the number of somites formed in a defined culture period. Blue bars denote DMSO treated samples and green bars denote RA treated samples. Error bars represent standard deviation. Abbreviations: som (somites) and tb (tail bud).

This onset of *Raldh2 *in the tail bud could in theory have an effect on the proliferative status of the tissue and the cessation of the growth of the tail bud. To support this possibility we first investigated if this onset is associated with detectable RA activity. To that end we tested for RA activity in the tail bud by co-culturing embryonic samples of tail bud from different developmental stages on top of a monolayer of F9 cells stably transfected with a 1.8 kb promoter sequence of a RA-responding element coupled to the *lacZ *gene (F9-1.8 cells) [55]. When we co-cultured F9-1.8 cells with tail bud explants from HH stage 20 embryos, which are *c-Raldh2*-negative, *lacZ *staining/RA activity was not detected (n = 7, Figure 5D), however, in the cultures incubated with tail bud explants from HH stage 21-26 embryos, which are *c-Raldh2*-positive, *lacZ *staining/RA activity was detectable (n = 8, Figure 5E, F). To confirm that changes in the levels of RA lead to changes in both the expression of Wnt/FGF markers and the growth of the chick tail we decided to artificially reproduce the onset of *Raldh2 *in the tail bud by grafting beads soaked in 1.5-10 mM RA adjacent to the tail bud of HH stage 12-15 chick embryos (n = 21) and then cultured them overnight for 12-15 hours. The treatment led to the extinction of both *c-Fgf8 *and *c-Wnt3a *expression in the tail bud (Figure 5H-J and data not shown; [45,47]) and also caused a significant reduction in PSM growth, as compared to control samples (t-test; df = 17, P < 0.001 for 1.5 mM RA and df = 20, P = 0.004 for 10 mM RA; Figure 5K, L). In summary, our results indicate that endogenous RA derived from a late onset of *Raldh2 *expression in the chick tail bud may be implicated in the late stages of somitogenesis/axial elongation by controlling the expression of Wnt and FGF factors in this domain.

Finally, we also analysed the expression of several relevant genes in a collection of 10.5-13.5 days post-coitum (dpc) mouse embryos to see if this onset of RA in the tail bud is conserved. We observed that *m-Wnt3a *is expressed in a broad area of the tail bud until E11.5 whereupon its expression is dramatically reduced to the very tip at E12.5 until it finally disappears at E13.5 (n = 14, Figure 4P-S). From E11.5 there is an onset of *m-Raldh2 *expression in the mouse tail bud (n = 19, Figure 4T-W), although it is noticeably weaker than that observed in chick, as judged by the intensity of the staining compared with the expression detected in the somites. In addition, when we evaluated the endogenous retinoic acid activity in mouse tail bud explants from E12.5 using the F9-1.8 cells we were not able to detect retinoid activity (data not shown). However, in the mouse embryo there is an alternative source of RA. The size of the mouse PSM is quite small [Additional File 2], thus the RA-producing somites are located close to the tail bud and may act as a source of RA, which could therefore be affecting this tissue during somitogenesis. Because of this proximity the tail bud region needs to be constantly protected from this source of RA and if this protection is lost, for example in *Cyp26A1*-/- embryos, then the tail bud becomes prematurely exposed to RA and the result is the generation of axial truncations [49-51]. Interestingly, we found that *m-Cyp26A1 *is reduced at E11.5 until it disappears (n = 16, Figure 4X-AA), suggesting that once the protective expression of *m-Cyp26A1 *is downregulated then RA may also affect the mouse tail bud in the mouse as a result of the onset of *m-Raldh2 *in the tail bud as well as *m-Raldh2 *from the somites.

## Discussion

We report here that termination of the process of somitogenesis seems to be the result of a combination of events occurring in the paraxial mesoderm tissue and the precursor population of the tail bud (see schematic representation in Figure 5G).

### Speed of somitogenesis and Wnt3a

Our data shows that the termination of somitogenesis is not the consequence of an acceleration of the speed of somite formation. In fact, this speed remains constant during the formation of all somites except for the last few, which require a longer time. For example, our data shows that at HH stage 23 somite formation might require 150 minutes. Why somite formation takes longer at those late stages of development is still unknown. We think it could be a collateral effect related to the diminution of *Wnt3a *in the tail bud at these stages, since we have previously shown that when Wnt activity is attenuated *in vitro *the period of *c-Lfng *oscillations is increased [33]. Interestingly, our data also indicates that there were consistently fewer somites formed in RA-treated embryos as compared to control embryos (Mann-Whitney Rank Sum Test; P = < 0.001 for 1.5 mM RA and P = 0.009 for 10 mM RA; Figure 5K, M), an effect that could be recapitulating *in vitro *the physiological slowing we observed at late stages of development (Figure 2), and which is coincident with the presence of RA activity in the tail bud and the concomitant downregulation of *Wnt3a *expression (Figure 4).

### The arrest of the somitic oscillator

The examination of the number of somites formed at each specific stage using as reference the expression of *Tbx6 *in the PSM and *Dact2/MyoD *in the forming somite shows that chick somitogenesis is completed (51-53 somites) at around HH stage 24-25, rather than at HH stage 22 [30]. Despite the fact that no further somites are formed, some *Tbx6*-positive PSM tissue remains at HH stage 24-25. This expression domain diminishes progressively until it disappears at HH stage 26-27. These observations indicate that the paraxial mesoderm does not become fully segmented to the tip of the tail, as indeed proposed by Bellairs and colleagues more than two decades ago [26,56]. Nevertheless, by HH stage 26-27 the last formed somite is positioned adjacent to the tail tip. The gradual shift of position of the last formed somite towards the tail tip may be due to the morphological re-shaping of the tail bud that happens at these stages of development [39].

There must be a reason why the terminal PSM does not segment. Sanders et al. proposed that this limitation could be imposed by different reasons, including the local environment and the existence of apoptosis [26]. *Tbx6 *expression within this tissue indicates that it maintains its PSM genetic character even after somitogenesis has concluded. The results clearly indicate that, as judged by the patterns of expression of components of the Notch pathway, the somitogenesis oscillator is arrested at these stages of development. Thus, the expression of Notch components, such as *c-Lfng*, is initially lost in the caudal PSM and becomes restricted to the rostral half of the PSM (HH stage 24) until it disappears (HH stage 25). This would suggest that the same tissue that displayed the last oscillations in the caudal PSM did so prior to HH stage 24, and as this tissue becomes displaced more rostrally it is also the last tissue to display non cyclic Notch activity implicated in boundary formation and thereby makes the last somitic border. We have recently shown that when all Notch activity is lost then somitogenesis is arrested [57]. The mechanism involved in the loss of Notch activity at these late stages and the subsequent arrest of the somitic oscillator is still unknown, although it is tempting to speculate that the downregulation of *Wnt3a *observed at HH stages 21-24 could also be implicated in this arrest because the expression of the ligand Delta1 in the caudal PSM has been shown to be dependent on Wnt3a, at least in the mouse embryo [58,59]. Also consistent with this hypothesis is the fact that when Wnt/FGF activities are temporally blocked after pharmacological treatment the expression of cyclic genes is arrested in the mouse [33,60]. It would be interesting to test this model during chick development by blocking Wnt/FGF activities long term and then analysing their morphological consequences.

### Last remaining PSM and apoptosis

Since the last few *Tbx6-*positive PSM cells do not contribute to make additional somites the question arises what becomes of them. TUNEL staining at HH stages 25-26 is restricted to the terminal region of the tail [Additional File 1] [37,38], which suggests that at least part of this remaining unsegmented paraxial mesoderm population could be dying by programmed cell death, as previously suggested [27]. This possibility would be consistent with the irregular pattern of expression of *Tbx6 *observed at HH stage 26. However, we cannot formally rule out the possibility that part of this population changes fate, loses *Tbx6 *and is incorporated in other neighbouring structures of the embryo. If current technical limitations can be overcome in the future, it would be very interesting to explore this possibility by following their fate.

### Endogenous RA and the tail bud

We describe here an onset of *Raldh2 *in both the chick and mouse tail bud that at least in the case of the chick embryo is strong enough to generate detectable levels of RA, as scored by *lacZ *staining with the F9-1.8 RA-responding cell line. The precise temporal correlation between the onset of *Raldh2 *expression and RA activity in the chick tail bud, and the observed downregulation of *Wnt3a *suggests these events could be linked, as has been shown to be the case after addition of ectopic RA [45-48]. In addition, as discussed above, we think that the longer period of the somitogenesis oscillator is likely to be an indirect effect of this RA-mediated *Wnt3a *downregulation.

If endogenous RA is also implicated in the cessation of segmentation in the mouse embryo by downregulation of *Wnt3a*/*Fgf8 *then the main source of this RA is probably the somites. In normal conditions the expression of *Cyp26A1 *in the mouse tail bud, which is regulated by trunk Hox (paralog group 13) and Cdx factors [61], prevents the diffusion of RA from the somites into the tissue and thereby prevents premature exposure of the progenitor population to RA. However, its removal in the *Cyp26A1*-/- mouse embryos allows endogenous RA to function unregulated in the mouse tail bud inducing dramatic abnormalities on the development of caudal structures including severe truncations [49-51]. The phenotype of this mouse mutant line is very similar to the sacral agenesis produced after teratological exposure to high levels of ectopic RA [62-65]. Thus, elevated retinoid activity, endogenous or ectopic, leads to a rapid and significant decrease in the expression of caudal markers, such as *Wnt3a *[45,46,48-50]. We think a similar elevated local retinoid activity, although highly regulated, could be affecting the tail bud in normal embryos at late stages of development due to the physiological downregulation of *Cyp26A1*, and which is concomitant with the onset of *Raldh2 *expression and the downregulation of *Wnt3a *and *Fgf8*.

### Open questions

The signals responsible for the growth of the precursor population seem to be reversible, since transplanting chick and mouse tail bud cells from a late stage embryo to earlier stages results in the grafted tissue contributing to somites along the entire axis, as well as re-colonising the host tail bud after culture [13,17,66]. These results indicate that termination of axial elongation is likely to depend upon changes in the tissue environment in the tail bud rather than a loss of competence to self-renew. Further analyses will be required to generate comprehensive answers concerning the nature of the molecular and cellular changes in the tail bud at late stages of somitogenesis. These analyses should provide a better understanding of how the somitogenesis oscillator is arrested at these stages of development, how the growth of the precursor population is affected and how relevant the exposure to endogenous RA is to this process. The degree of conservation of each of these mechanisms in different species, and how they are each regulated will also contribute to explain the generation of the very varied and complex body plans displayed by different vertebrate species.

## Conclusions

Our results show that the termination of the process of somitogenesis in the chick embryo is not due to acceleration in the speed of somite formation because it remains constant, except at late stages of development when it slows down. We found that when the chick embryo reaches its final number of somites at stage HH 24-25 there is still some remaining unsegmented presomitic mesoderm, in which the expression of components of the somitogenesis oscillator is no longer dynamic, suggesting that at these stages of development the somitic oscillator is arrested. Finally, endogenous RA emanating from an onset of expression in the tail bud and/or from the somites is probably involved in the control of the process of somitogenesis, and in tail growth in general, by controlling the expression of Wnt3a and Fgf8.

## Methods

### Embryos and *in situ *hybridisation

White leg horn *Gallus gallus *eggs were sourced from Henry Stewart & Co (Lincolnshire) and incubated at 38.5°C in a humidified incubator. Embryos were staged according to the Hamburger Hamilton (HH) developmental table [30] and by somite counting. Wild-type CD1 mouse (*Mus musculus) *embryos were obtained from timed mated pregnant females between 10.5 and 13.5 dpc (E10.5-E13.5). *In situ *hybridisation analysis was basically performed as described [67]. Proteinase K treatment for old chick embryos was as follows: HH stage 20, 20.5 min; HH stage 21-22, 22 min; HH stage 23-24, 24.5 min; HH stage 24-25, 26 min, HH stage 26-27, 28 min, HH stage 28, 29 min. Images were captured on a Leica MZ16 APO microscope using a Jenoptik camera. All animals were handled in strict accordance with good animal practice as defined by the Home Office (UK) and local animal welfare bodies, and all animal work was approved by the ethical committee for experiments with animals of the University of Dundee (UK).

### Somite formation curve and PSM size measurements

Eggs (n = 105) were incubated at 38.5°C until they reached HH stage 8 (3-5 pairs of somites), then carefully windowed and the shell membrane above the embryo was removed and ink diluted 1:10 with Phosphate-buffered saline (PBS) was injected underneath the embryo to visualize the somites. The initial number of somites was counted. Eggs were then sealed with parafilm and incubated at 38.5°C for specific time periods and the final number of somites was recounted. For the latest stages (HH stage 23-26), somites were visualized and counted after *cDact2/cMyoD *double *in situ *hybridisation. The PSM was measured from the posterior boundary of the last formed somite to the end of the neural tube. The size of the PSM and of the last formed somite was measured using Carl Zeiss AxioVision 4 software and a grid of the Neubauer Improved counting chamber as a standard for the measurements.

### Apoptosis and manipulation of the Retinoid pathway

For detection of apoptotic cells, the ApopTag Plus Peroxidase *In situ *kit (Chemicon) was used. For ectopic RA treatment 1.5-10 mM all-trans-RA or Dimethyl sulfoxide (DMSO) soaked beads were implanted *in vivo *in the tail bud region of HH stage 12-15 embryos, which were then incubated overnight before collecting the embryos for analysis. For RA detection F9 cells transfected with a 1.8 kb promoter sequence of the mouse retinoic acid β2 receptor coupled to the *lacZ *gene were grown to confluence as described [55]. Chick embryos were dissected in PBS and the tail bud tip or last formed somites were isolated and placed on top of RA-responding cells for 10 minutes to allow their attachment. 0.5 ml media was added and the cultures were incubated overnight at 38.5°C in a humidified incubator with 5% CO_2_. Alternatively, a solution containing 1-10 nM RA was added in place of the explants as a control for detection of RA activity. *LacZ *activity was detected by X-Gal staining overnight at 37°C performed according to the protocol for β-Galactosidase reporter gene staining kit (Sigma).

## Authors' contributions

MM designed the project and the experiments. GT, DW, ZF, MMc, RB and MM performed the experiments. GT, DW, ZF and MM analysed the data. MM wrote the manuscript and prepared the figures. All authors read and approved the final manuscript.

## Supplementary Material

Additional file 1 **Supplementary Figure 1; Apoptosis in the chick tail bud**. (A, B) Dorsal and (C-F) lateral views of chick embryos analysed by TUNEL staining to identify cell death in the chick tail bud at HH stages 13-26, showing there strong localised apoptosis in the tail bud of HH stage 13-19 and then again in the terminal region at HH stage 26. Click here for file

Additional file 2 **Supplementary Figure 2; *Wnt3a *diminution and RA activity in the mouse tail bud**. (A-D) Lateral view of E10.5-E13.5 mouse tail buds analysed by *in situ *hybridisation using *m-Tbx6*. (E) Bar chart showing the size of the PSM (green bars) and the size of the last somite (red bars) at E10.5-13.5. (F) Bar chart showing the ratio PSM versus last somite during the same window of development calculated separately for each embryo and then the average ratio was calculated for each stage. Error bars represent standard deviation. Black dotted lines represent the variation of these parameters observed in the chick embryo from HH stage10 to stage 25, as shown in Figure 1. Click here for file
