# Supplementary figures and images for: The chick somitogenesis oscillator is arrested before all paraxial mesoderm is segmented into somites
Source: BMC Dev Biol. 2010 Feb 25;10:24. doi: 10.1186/1471-213X-10-24 (PMC2836991; doi:10.1186/1471-213X-10-24)

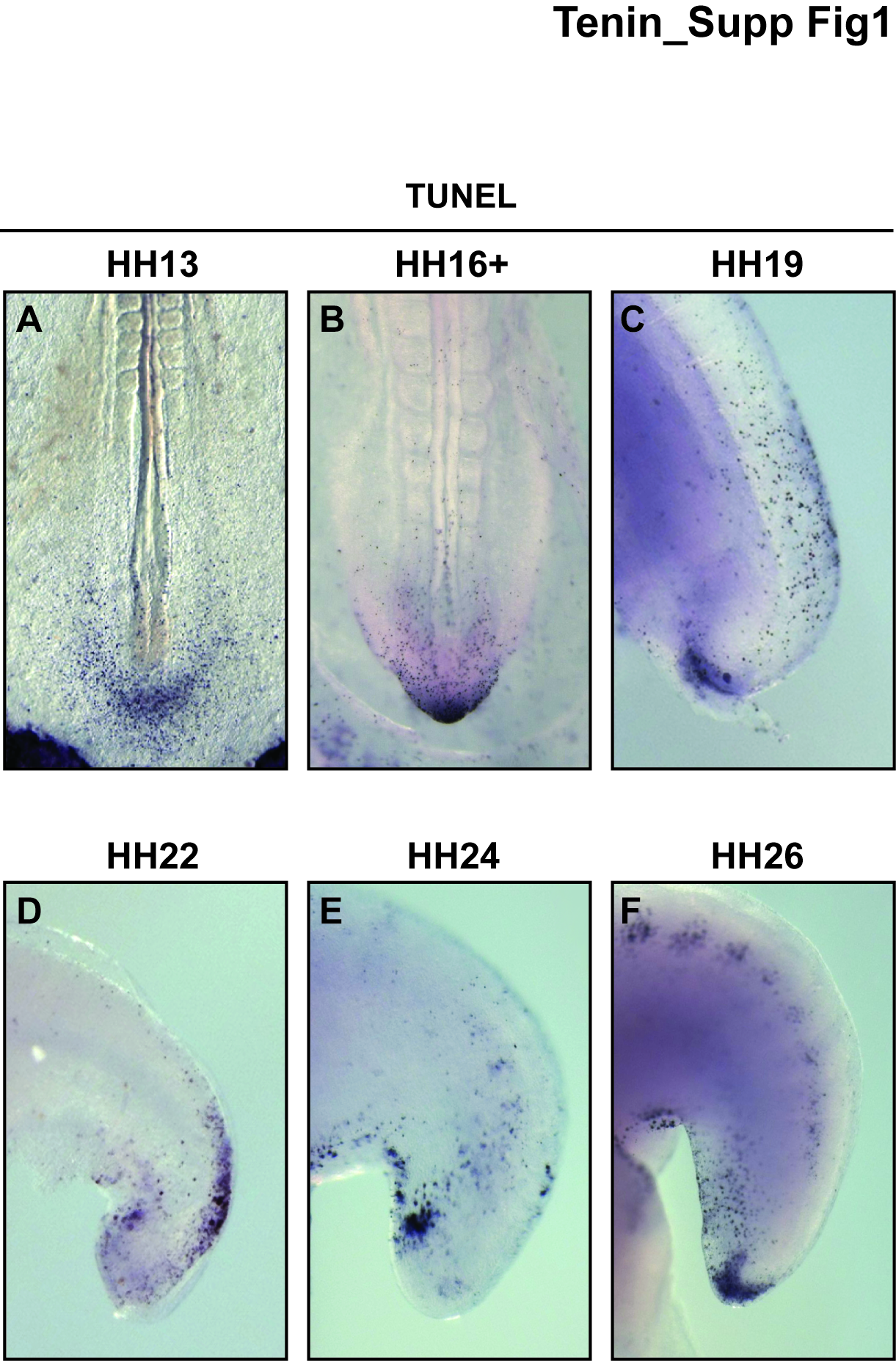

Supplement: Additional file 1 — Supplementary Figure 1; Apoptosis in the chick tail bud. (A, B) Dorsal and (C-F) lateral views of chick embryos analysed by TUNEL staining to identify cell death in the chick tail bud at HH stages 13-26, showing there strong localised apoptosis in the tail bud of HH stage 13-19 and then again in the terminal region at HH stage 26. [file 1471-213X-10-24-S1.TIFF]

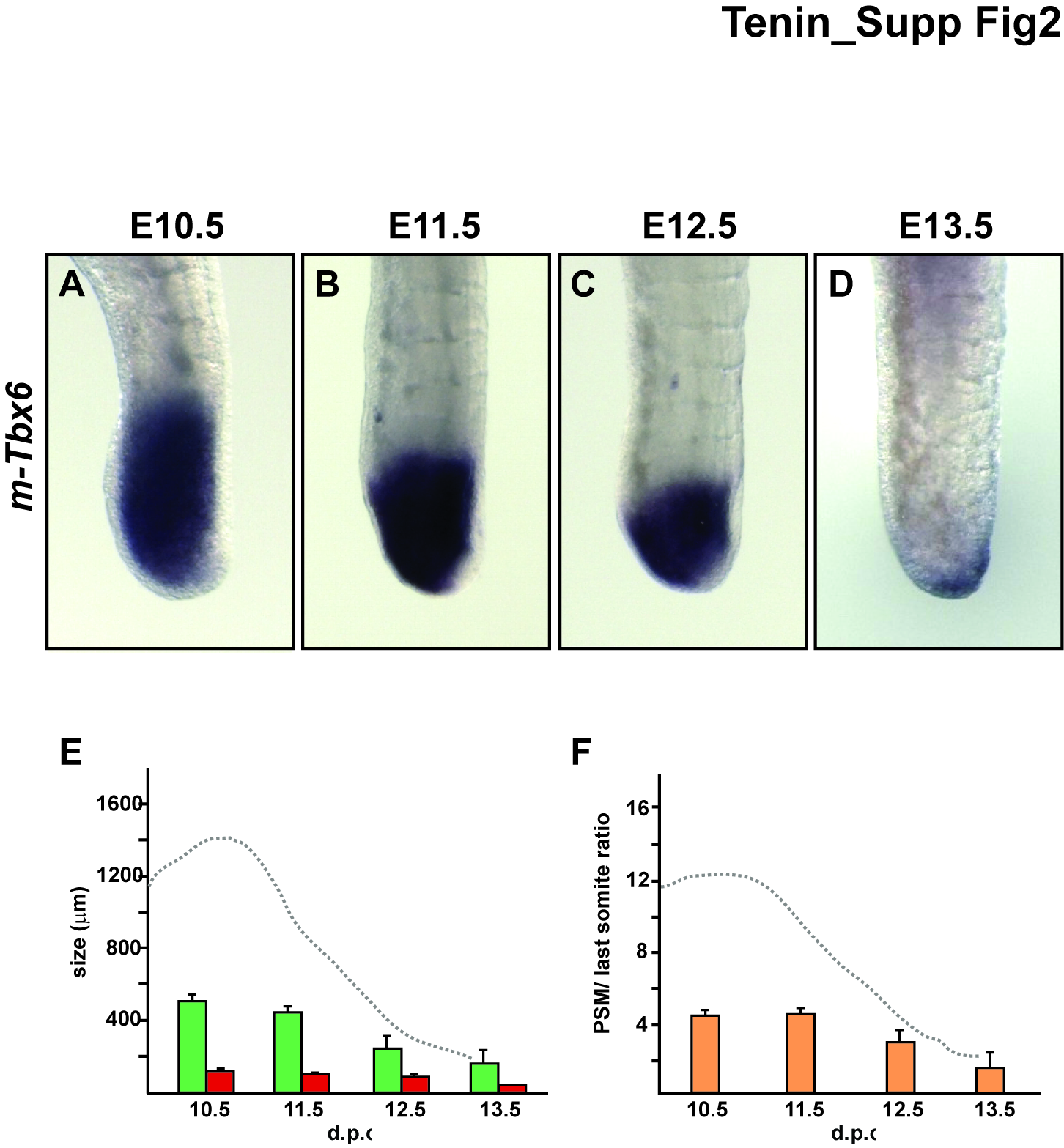

Supplement: Additional file 2 — Supplementary Figure 2; Wnt3a diminution and RA activity in the mouse tail bud. (A-D) Lateral view of E10.5-E13.5 mouse tail buds analysed by in situ hybridisation using m-Tbx6. (E) Bar chart showing the size of the PSM (green bars) and the size of the last somite (red bars) at E10.5-13.5. (F) Bar chart showing the ratio PSM versus last somite during the same window of development calculated separately for each embryo and then the average ratio was calculated for each stage. Error bars represent standard deviation. Black dotted lines represent the variation of these parameters observed in the chick embryo from HH stage10 to stage 25, as shown in Figure 1. [file 1471-213X-10-24-S2.TIFF]
